# Genetic clustering and polymorphism of the merozoite surface protein-3 of *Plasmodium knowlesi* clinical isolates from Peninsular Malaysia

**DOI:** 10.1186/s13071-016-1935-1

**Published:** 2017-01-03

**Authors:** Jeremy Ryan De Silva, Yee Ling Lau, Mun Yik Fong

**Affiliations:** Department of Parasitology, Faculty of Medicine, University of Malaya, 50603 Kuala Lumpur, Malaysia

**Keywords:** *Plasmodium knowlesi*, Merozoite surface protein-3, Genetic diversity, Natural selection, Haplotypes

## Abstract

**Background:**

The simian malaria parasite *Plasmodium knowlesi* has been reported to cause significant numbers of human infection in South East Asia. Its merozoite surface protein-3 (MSP3) is a protein that belongs to a multi-gene family of proteins first found in *Plasmodium falciparum*. Several studies have evaluated the potential of *P. falciparum* MSP3 as a potential vaccine candidate. However, to date no detailed studies have been carried out on *P. knowlesi* MSP3 gene (*pkmsp3*). The present study investigates the genetic diversity, and haplotypes groups of *pkmsp3* in *P. knowlesi* clinical samples from Peninsular Malaysia.

**Methods:**

Blood samples were collected from *P. knowlesi* malaria patients within a period of 4 years (2008–2012). The *pkmsp3* gene of the isolates was amplified *via* PCR, and subsequently cloned and sequenced. The full length *pkmsp3* sequence was divided into Domain A and Domain B. Natural selection, genetic diversity, and haplotypes of *pkmsp3* were analysed using MEGA6 and DnaSP ver. 5.10.00 programmes.

**Results:**

From 23 samples, 48 *pkmsp3* sequences were successfully obtained. At the nucleotide level, 101 synonymous and 238 non-synonymous mutations were observed. Tests of neutrality were not significant for the full length, Domain A or Domain B sequences. However, the dN/dS ratio of Domain B indicates purifying selection for this domain. Analysis of the deduced amino acid sequences revealed 42 different haplotypes. Neighbour Joining phylogenetic tree and haplotype network analyses revealed that the haplotypes clustered into two distinct groups.

**Conclusions:**

A moderate level of genetic diversity was observed in the *pkmsp3* and only the C-terminal region (Domain B) appeared to be under purifying selection. The separation of the *pkmsp3* into two haplotype groups provides further evidence of the existence of two distinct *P. knowlesi* types or lineages. Future studies should investigate the diversity of *pkmsp3* among *P. knowlesi* isolates in North Borneo, where large numbers of human knowlesi malaria infection still occur.

**Electronic supplementary material:**

The online version of this article (doi:10.1186/s13071-016-1935-1) contains supplementary material, which is available to authorized users.

## Background

Malaria is a disease caused by the infection of blood protozoa belonging to the genus *Plasmodium*. Molecular evidence suggests that the simian malaria agent *Plasmosium knowlesi* evolved from a group which included *Plasmodium cynomolgi* and *P. vivax* some 30.5 million years ago [1]. The first report of natural transmission of *P. knowlesi* to humans was reported in 1965 when a US Army surveyor acquired the infection while working in Peninsular Malaysia [2]. It was observed that the parasite could be transmitted to humans through blood inoculation and thus the authors designated it the human strain or strain H. A second case was reported in southern Peninsular Malaysia 5 years later [3]. A large number of human knowlesi malaria was reported in Malaysian Borneo in 2004 [4], and reports have also been published on this infection in several neighbouring Asian countries such as Singapore [5], the Philippines [6] and Thailand [7]. However, the majority of the infections have been recorded in Malaysia. More than 300 human cases have been detected in Peninsular Malaysia since 2005 [8–10]. Recently, a study reported that more than half of the malaria cases in Malaysia were caused by *P. knowlesi* [11]. The highest proportion of *P. knowlesi* cases was found to be in the Malaysian Borneo as well as in the Peninsular Malaysia states of Kelantan, Pahang, Terengganu and Johor [11].

Malaria parasites invade the red blood cells (RBC) of many vertebrate hosts including humans and simians. The proteins involved in the invasion process have been studied to gain deeper insights of the invasion mechanism, and also to identify potential vaccine candidates against malaria [12]. One of these proteins, the merozoite surface protein-3 (MSP3), was identified in *P. falciparum* in 1994 [13, 14]. Subsequently, a novel surface antigen was discovered in *P. vivax* and was named MSP3α, due to its putative similarity to the MSP3 of *P. falciparum* [15]. Two paralogs of the *P. vivax* MSP3 protein were further identified, designated as PvMSP3β and PvMSP3γ [16]. Due to the presence of more than one such protein in a species, the *P. vivax* MSP3 proteins were grouped into a multi-gene family [17]. Full genome analysis on *P. vivax* (Salvador I strain) revealed 12 *msp3* paralogs which cluster on chromosome 10 [18]. Surprisingly, these paralogs have limited similarity to the *P. knowlesi* MSP3 and the four *P. falciparum* MSP3 proteins. Although a number of studies have suggested that the *msp3* genes in *P. vivax* and *P. falciparum* are related, a closer comparison between the domain organizations on chromosome 10 as well as the syntenic loci of *pvmsp3*, *pfmsp3* and *P. knowlesi* putative *msp3* genes suggest that these genes are not homologues [19].

Structurally the protein is characterized by a putative signal peptide and lacks a transmembrane domain or a GPI-lipid modification to anchor it to the outer membrane of the parasite. Another characteristic of the protein family is the presence of an alanine-rich central domain containing a series of heptad coiled-coil repeats [15, 20]. Recent studies have predicted that the MSP-3 proteins in *P. vivax* form oligometric and elongated molecules suggesting the protein may mediate interactions between host proteins and other merozoite surface proteins [21].

Genetic diversity in a natural population is usually generated by the introduction of new alleles through the process of migration, mutation, or recombination [22]. The frequency of these alleles on the other hand is governed by the actions of selection and natural drift [23]. For pathogens that infect humans, the host’s immune responses as well as modes of treatment administered are major components of selection, thus, genetic diversity can be an important indicator of how a pathogen responds to modes of intervention such as vaccines or drugs [24]. In this instance, directional selection leads towards fixing beneficial alleles in the population, resulting in reduced diversity [25]. Conversely, naturally acquired host immunity can exert balancing selection which tends to preserve or increase the allelic diversity of antigen genes. This of course occurs within the functional constraints of the encoded protein to prevent the protein from losing its native ability and function [26, 27]. The modelling of neutral processes in a population with a constant size allows for the prediction of expected frequencies of a particular allele. Thus, departures from this neutrality can thus be utilised to identify or pinpoint alleles that are targets for directional or balancing selection [28–31].

Several studies have been carried out on MSP3 proteins of *P. falciparum* and *P. vivax*; however, studies on *P. knowlesi* MSP3 lag far behind. In this study, the genetic diversity, natural selection and haplotype groups of *pkmsp3* gene of *P. knowlesi* clinical isolates from Peninsular Malaysia were studied. Evidence of purifying selection in the C-terminal domain and haplotype grouping of *P. knowlesi* MSP3 was found. These data will be useful in understanding the genetic variation and natural selection forces acting on this gene and may indicate the gene’s potential as a vaccine candidate.

## Methods

### Blood sample collection

Twenty-three blood samples from knowlesi malaria patients were obtained from the University of Malaya Medical Centre (UMMC), Kuala Lumpur as well as from private clinics in Peninsular Malaysia between July 2008 and July 2012. Each blood sample was assigned a reference code for laboratory record. Knowlesi malaria infection was re-confirmed using several tests including microscopic examination of Giemsa-stained thick and thin blood smears, BinaxNOW® malaria rapid diagnostic test (Inverness Medical International, Stockport, United Kingdom) and polymerase chain reaction (PCR) based on the *Plasmodium* small subunit ribosomal RNA gene [4].

### Genomic DNA extraction

Genomic DNA was extracted from the blood samples using a commercial blood extraction kit (QIAGEN, Hilden, Germany). One hundred μl of blood were used per extraction and the DNA was eluted into 50 μl of TE Buffer.

### PCR, cloning and sequencing of *pkmsp3*

The *pkmsp3* gene was amplified by nested PCR. For the initial primary PCR, oligonucleotide primers MSP3N1F: 5′-CCT CTT CAA CCA CAC ACA CA-3′ and MSP3N1R: 5′-GTT CAT TCT GGC GGA TAA GG-3′ were used [19]. Oligonucleotide primers MSP3N2F: 5′-CCC GTG AAA TAA CAC CCA-3′ and MSP3BN2R: 5′-CCA CCA TCT TAC GTT CAG-3′ [19] were used for the secondary PCR. Approximately 0.5 μg of genomic DNA was used in a final volume of 20 μl which also contained 0.2 mM of dNTP, 0.4 μM of forward and reverse primers, 2 mM MgCl_2_ and 1 unit of *Taq* DNA polymerase in buffer provided by the commercial kit (Promega, Madison, WI, USA). The PCR thermal profile was as follows, an initial denaturation of one cycle at 95 °C for 5 min followed by 30 cycles of 1 min at 94 °C, 1 min at 50 °C for annealing and 1 min 30 s at 72 °C for nest 1. Cycling for nest 2 consisted of a 5 min initial denaturation at 95 °C and 30 cycles of 1 min at 94 °C, 1 min at 48 °C, 1 min 30 s at 72 °C, and a final extension step at 72 °C for 10 min. PCR products were analysed by gel electrophoresis on a 1.5% agarose gel stained with SYBR® Safe DNA gel stain (Invitrogen, Eugene, USA).

### Purification of PCR products and DNA cloning

PCR products were purified using QIAquick PCR purification kit (Qiagen, Hilden, Germany) per the manufacturer’s instructions. The purified PCR products were then ligated into the pGEM-T® TA cloning vector (Promega, Madison, Wisconsin, USA) and transformed into *Escherichia coli* TOP10F’ competent cells; colonies were then screened for the presence of recombinant plasmids harbouring the *pkmsp3* fragment. These plasmids were then sequenced in a commercial laboratory (MyTACG Bioscience Enterprise, Malaysia). Between 3 and 5 recombinant plasmids were sent for sequencing per isolate. DNA for isolates showing clonal sequence variations (singletons or rare substitutions) was re-amplified and re-sequenced in order to confirm that the variations were genuine, and not the result of incorporation errors of the *Taq* DNA polymerase.

### Analysis of *pkmsp3* sequences

Editing and alignment of the *pkmsp3* nucleotide sequences (including the sequence of reference *P. knowlesi* strain H, GenBank: XM_002259752) were performed using the BioEdit sequence alignment editor ver. 7.2.0. Gene Runner ver. 4.0.9.2 was used to deduce the respective amino acid sequences. The Neighbour Joining method described in MEGA6 was used to construct a phylogenetic tree [32] with bootstrap replicates of 1000. The Median-Joining method in NETWORK v4.6.1.2 program [33] was used to establish the genetic relationship among *pkmsp3* haplotypes and construct the haplotype network. All newly-generated sequences were deposited in the GenBank database (KT900798–KT900845).

### Sequence polymorphism analysis of *pkmsp3*

The programme DnaSP ver. 5.10.01 [34] was used to determine *pkmsp3* genetic polymorphism by calculating the number of nucleotide differences per site (π), singleton sites (S), segregating sites (Ss), haplotypes (H), parsimony-informative sites (Ps), and haplotype diversity (Hd) [35].

The neutral model of molecular evolution acting on the *pkmsp3* was tested according to nucleotide polymorphisms and haplotype distribution in the Fu and Li’s D* and F* tests [36]. The Tajima’s D test [22] was calculated to test the hypothesis that all mutations are selectively neutral. Tajima’s D test is based on the difference between Ss and π where positively significant values indicate balancing selection and negatively significant values indicate directional or purifying selection. In all tests carried out, sites that had gaps were excluded from the analysis. In tests requiring an outgroup, *Plasmodium cynomolgi* MSP3 was used (GenBank: KC907504). The F_ST_ fixation index [37] in DnaSP 5.10.00 was used to measure the genetic differentiation between the different clustering groups observed in the *pkmsp3* phylogenetic tree and haplotype network.

The effect of natural selection was evaluated by the codon based *Z*-test, which determines whether it is negative or positive selection. Probability (*P*) values of less than 0.05 were considered significant. The variance of the differences was computed using the bootstrap method with 1000 replicates. The ratio between the average number of non-synonymous substitutions per non-synonymous site (d_N_) and the average number of synonymous substitutions per synonymous site (d_S_) using the Nei-Gojobori method with Jukes and Cantor correction [38] was also calculated. MEGA6 was used to calculate the *Z*-test and d_N_/d_S_ ratio [32].

**Fig. 1:**
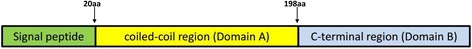
Domain structures in *pkmsp3*. Organisation of the *pkmsp3* gene showing the positions of coiled-coil region identified as Domain A (*yellow*), the C-terminal region as Domain B (*blue*) and the signal peptide (*green*)

The Interpro programme (http://www.ebi.ac.uk/interpro) predicted the *P. knowles*i MSP3 to have a large coiled-coil region. Genetic diversity and selection analyses were also performed separately on the coiled-coil region (Domain A) and the C-terminal (Domain B) of the protein (Fig. 1). This was carried out to investigate domain specific selective pressure.

## Results

### Genetic diversity at the nucleotide level

Successful PCR amplification produced DNA fragments of 1077 bp. This fragment contained a region coding a protein sequence of 338 amino acids. A total of 48 sequences were obtained for analysis.

Table 1 gives the estimates of genetic diversity for the full length *pkmsp3* sequence, Domain A and Domain B. In the full length sequence, 384 segregating sites were observed; of these, 320 were parsimony-informative and 64 were singleton sites. When separated into Domain A and B, however, Domain B contained more segregating sites as compared to Domain A (273 *vs* 104). As for diversity, the full length sequence had haplotype diversity (Hd) of 0.997 ± 0.005. Both Domains A and B had similar Hd of 0.989 ± 0.007.

**Table 1:** Estimates of DNA diversity, selection, and neutrality tests of full length, Domain A and Domain B of *pkmsp3* gene

*Pkmsp3*	Sites^a^	Ss	S	Ps	Hd ± SD	π ± SD	d_N_ ± SE	d_S_ ± SE	d_N_/d_S_	*Z*-test *P*-values			Tajima’s D	Fu & Li’s	
										d_N_ = d_S_	d_N_ > d_S_	d_N_ < d_S_		D*	F*
Full length	1,077	384	64	320	0.997 ± 0.005	0.046 ± 0.011	0.052 ± 0.004	0.058 ± 0.008	1.1	0.45	1.00	0.24	-1.440	-0.077	1.046
Domain A	534	104	44	60	0.989 ± 0.007	0.039 ± 0.002	0.039 ± 0.002	0.030 ± 0.008	1.3	0.34	0.10	1.00	-0.723	-1.852	-1.670
Domain B	483	273	16	257	0.989 ± 0.007	0.067 ± 0.025	0.025 ± 0.007	0.042 ± 0.002	0.6	0.19	1.00	0.09	-1.918	-1.579	-1.711

*Abbreviations*: *d*_*N*_ non-synonymous polymorphism, *d*_*S*_ synonymous polymorphism, *d*_*N*_ */d*_*S*_ ratio of d_N_ to d_S_, *Hd* haplotype diversity, *Ps* number of informative-parsimonious sites, *π* nucleotide diversity, *S* number of singleton sites, *SD* standard deviation, *Ss* number of segregating sites

^a^Total number of sites analysed excluding gaps

*Modified Fu & Li’s D and F tests

**Fig. 2:**
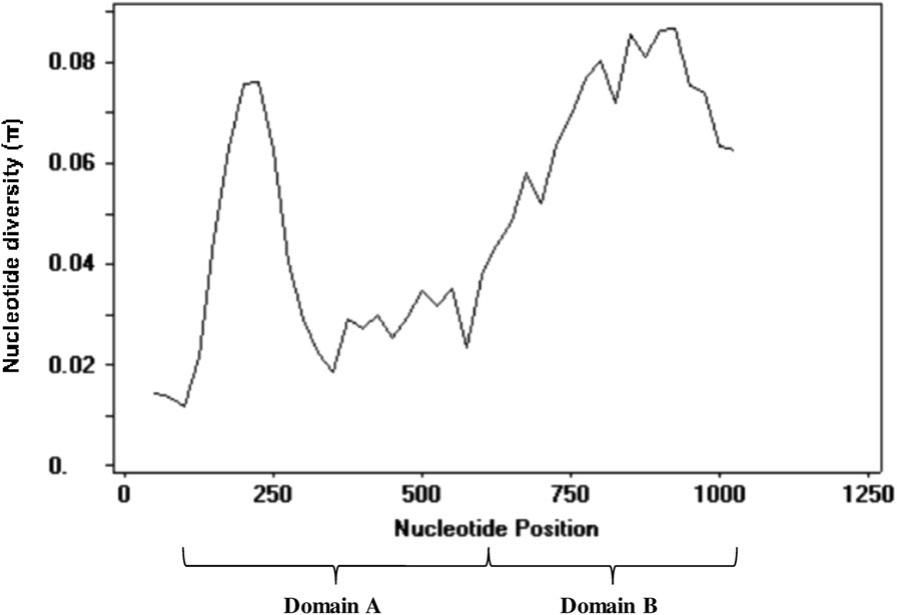
Nucleotide polymorphism in the *pkmsp3*. Sliding window plot of the nucleotide diversity (π) along the *pkmsp3*, generated with a window length of 100 bp and step size of 25 bp

Nucleotide diversity (π: 0.046 ± 0.011) for the full length sequence was found to be several times higher compared to other *P. knowlesi* functional genes such as PkDBPαII (π: 0.012) [39], PkAMA-1 (π: 0.00501) [40] and PkRAP-1 (π: 0.01298) [41]. Diversity for Domain B (π: 0.067 ± 0.025) was found to be higher than that for Domain A (π: 0.039 ± 0.002). A sliding window plot with a window length of 100 bp and a step size of 25 bp provided a detailed analysis of the full length sequence, with π ranging from 0.012 to 0.087 (Fig. 2). The highest peak diversity was within nucleotide positions 801–975 in Domain B, whereas in Domain A, the most conserved region was within nucleotide positions 51–150.

### Genetic diversity at the amino acid level

**Fig. 3:**
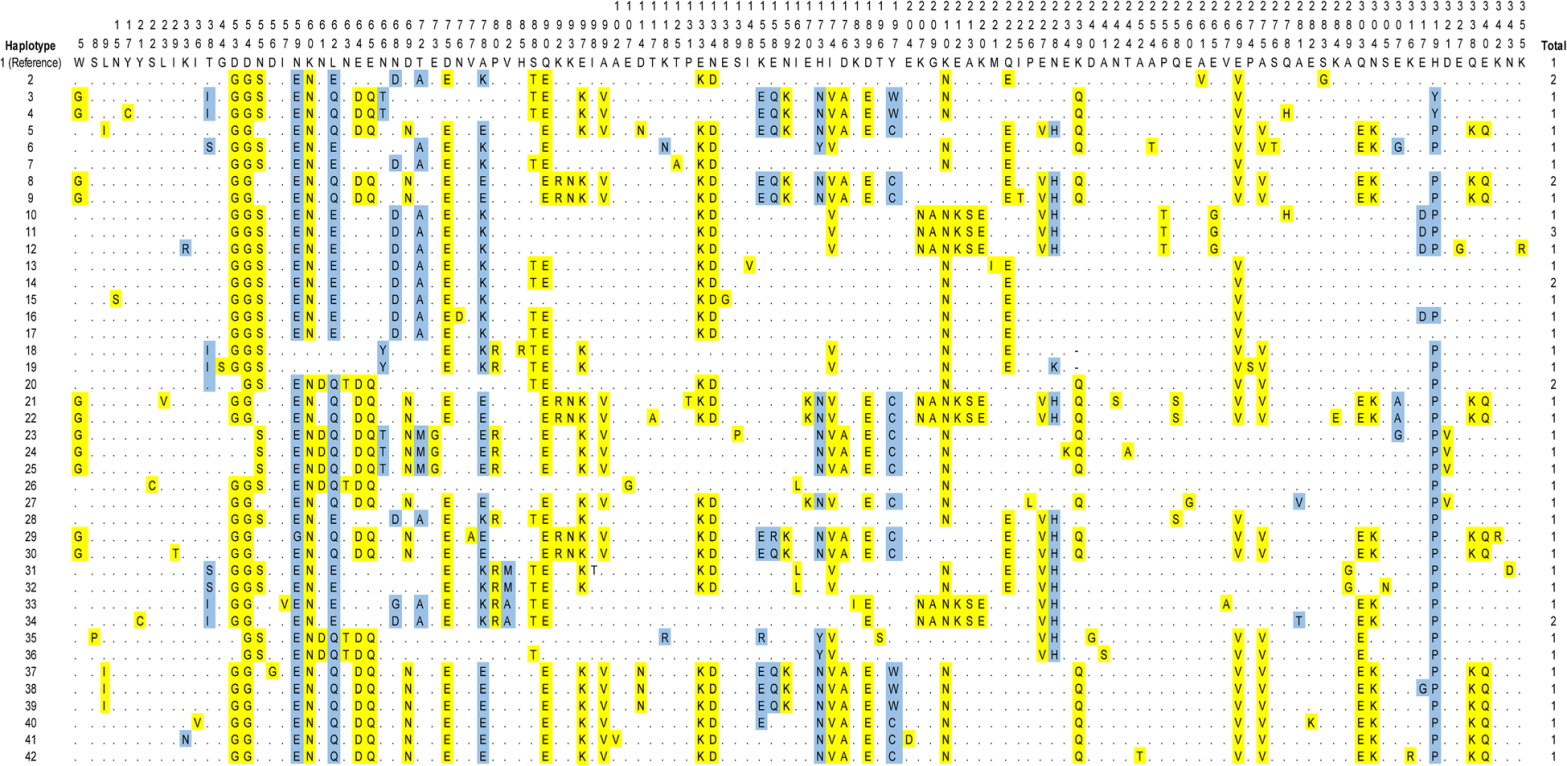
Amino acid sequence polymorphism in *pkmsp3*. Polymorphic amino acid residues are listed for each haplotype. Monomorphic and dimorphic amino acid changes are marked in *yellow* and *blue*, respectively. Total number of sequences for each haplotype is listed in the panel on the right

Comparisons and analysis with *P. knowlesi* strain H as a reference sequence showed mutations at 339 positions. Of these positions, 101 were synonymous changes and 238 were non-synonymous. When translated into deduced amino acids, high level polymorphism was observed (Fig. 3 and Additional file 1: Table S1). Among the 119 polymorphic sites, 100 were monomorphic mutations with a change into one amino acid type, and 19 showed dimorphic mutations with change in two amino acid types (K33R/N, T38I/S, N59E/G, L62E/Q, N66T/Y, N68D/G, T72A/M, A78K/E, V82M/A, K118N/R, K155E/R, E158Q/R, H173N/Y, Y197W/C, N228H/K, A281V/T, E307G/A, E317D/G and H319Y/P). The amino acid sequences could be categorised into 42 haplotypes (H1-42) (Fig. 3) with haplotype 11 having the highest frequency. Fifteen of the 23 patient samples had mixed haplotype infections (Table 2).

**Table 2:** Haplotypes of *pkmsp3* detected in human blood samples. Each blood sample was assigned a reference code (alphabetical or numerical)

Blood sample code	Haplotype detected
ANU	H2
AZI	H3, H4
CHO	H5
GAN	H6
MAH	H7
NGO	H8, H9
OTH	H10, H11
RAU	H11, H12
SAM	H13, H14
SYA	H15, H16, H17
UM0001	H18, H19
UM0004	H20
UM0006	H21, H22
UM0009	H23, H24, H25
UM0014	H26
UM0015	H27
UM0016	H28
UM0018	H29, H30
UM0020	H31, H32
UM0029	H33, H34
UM0032	H35, H36
UM0047	H37, H38, H39
UM0050	H40, H41, H42
*P. knowlesi* strain H	H1

### Phylogenetic analysis of *pkmsp3*

**Fig. 4:**
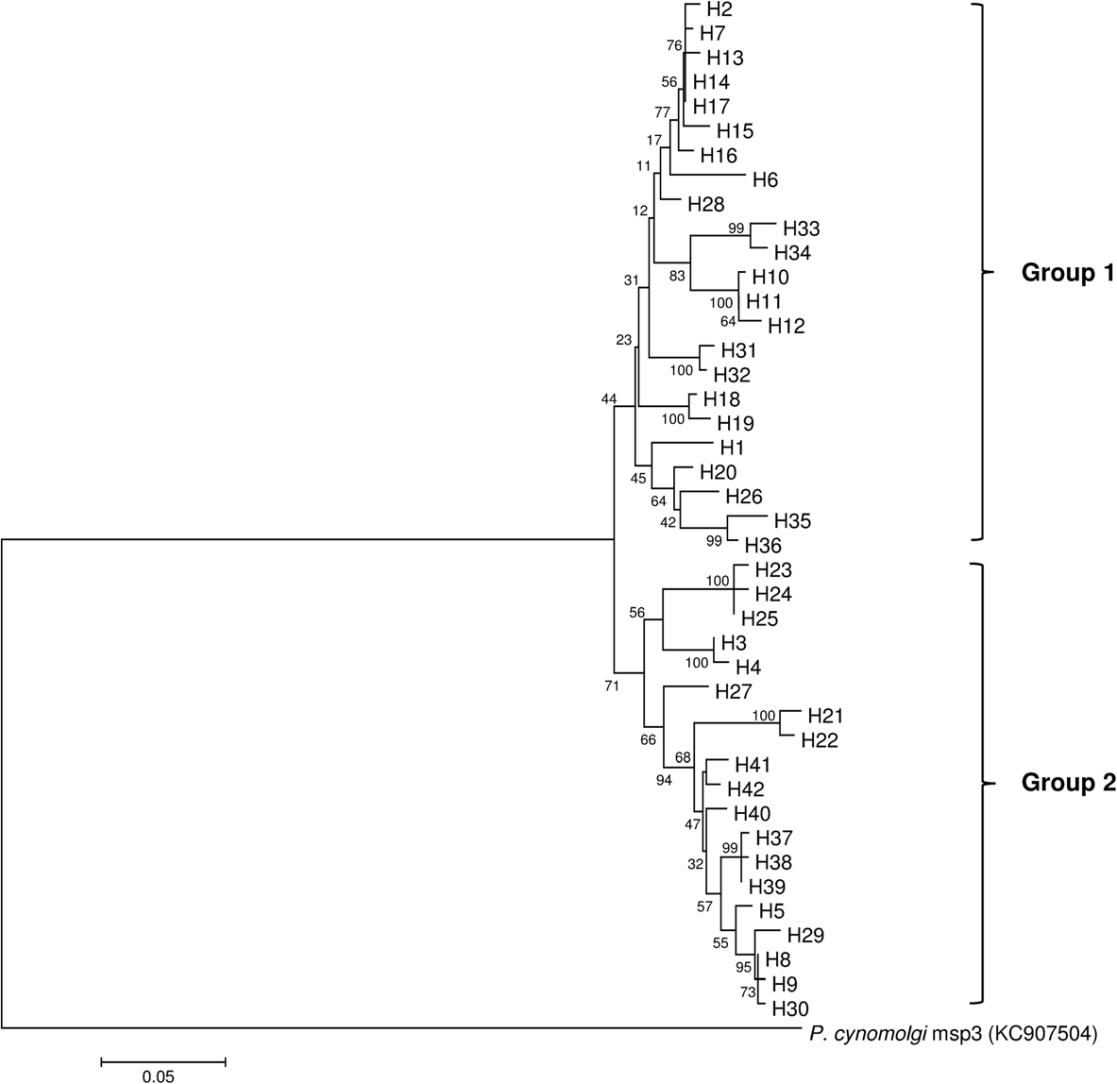
Phylogenetic tree of *pkmsp3* haplotypes. The neighbour joining method was used to construct the tree, which contains 42 haplotypes. Numbers at the nodes indicate percentage support of 1000 bootstrap replicates

**Fig. 5:**
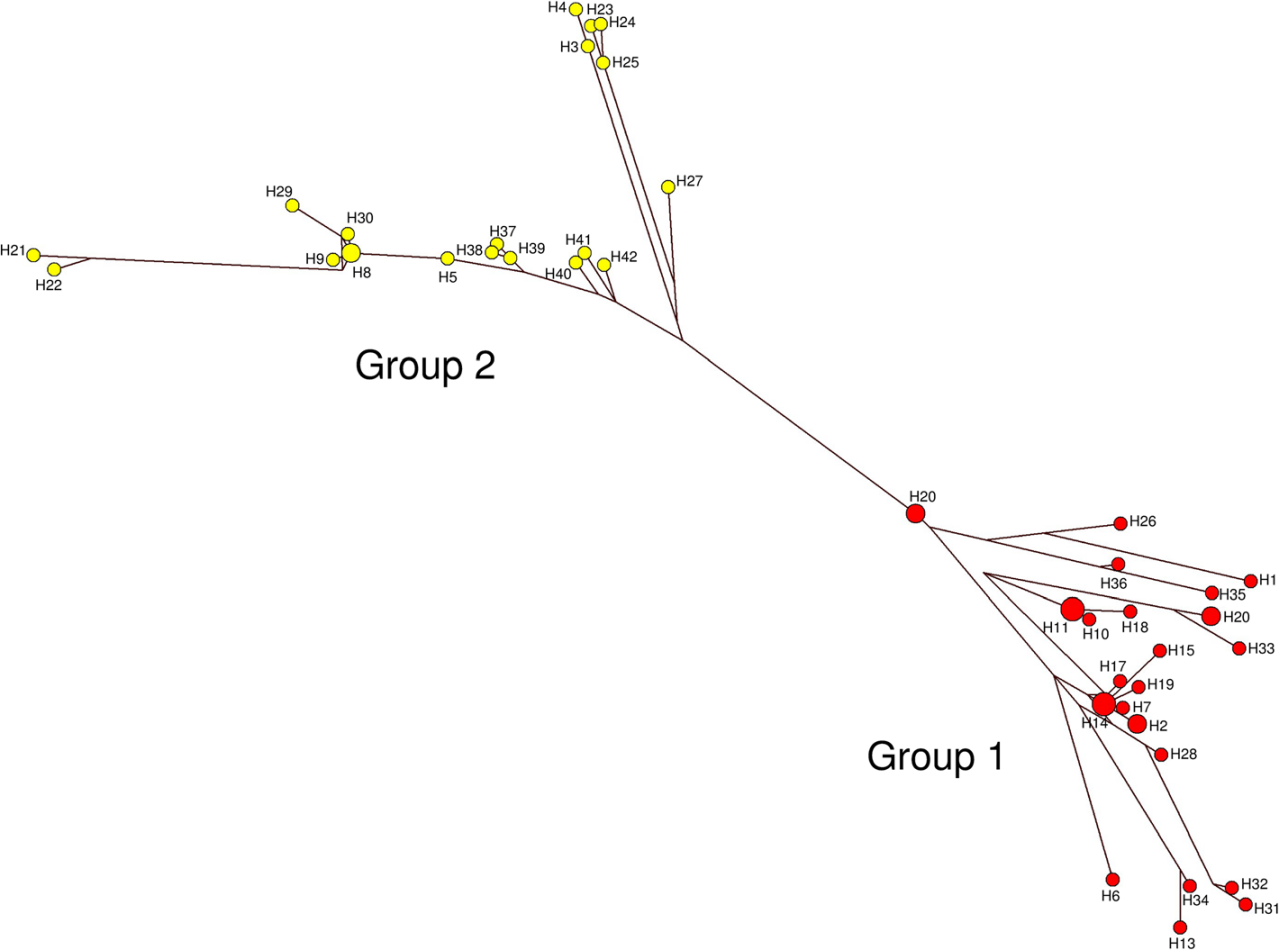
Network analysis of *pkmsp3* haplotypes. The NETWORK program v4.6.1.2 was used to construct the haplotype network, which contains 42 haplotypes. Nodes in *red* indicate Group 1 haplotype members and nodes in *yellow* indicate Group 2 haplotype members

Analysis of the phylogenetic tree (Fig. 4) and haplotype network (Fig. 5) revealed that the haplotypes are clustered into two main groups (Group 1 and Group 2), which contained almost equal number of haplotypes. Furthermore, mixed haplotypes from the same blood sample were found to cluster into the same group in both the phylogenetic tree (Fig. 4) and haplotype network and (Fig. 5).

**Fig. 6:**
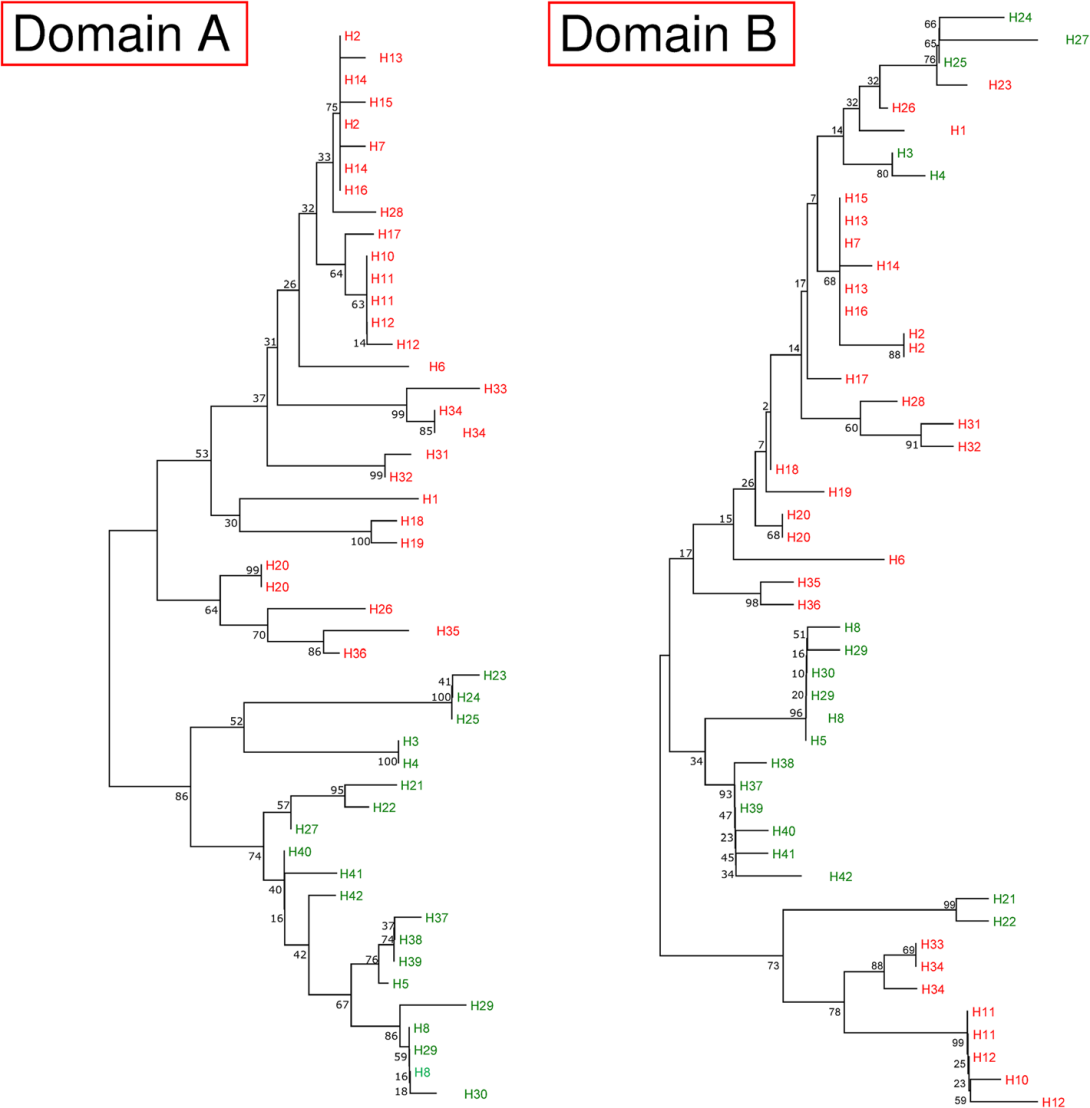
Phylogenetic trees of Domains A and B of *pkmsp3*. Neighbour joining method was used to construct the tree. In both trees, taxa indicated in *red* represent haplotypes of Group 1, whereas the taxa indicated in *green* are members of Group 2. The Domain A tree shows clustering similar to the tree of full length *pkmsp3* (Fig. 4). Numbers at the nodes indicate percentage support of 1000 bootstrap replicates

Further analysis was carried out to determine if Domain A or Domain B contributed to the haplotype clustering. A Neighbour Joining tree was constructed for both the domains (Fig. 6) and it was observed that polymorphisms in Domain A contributed to the haplotype clustering, as the clustering observed in this domain mirrored the tree constructed using the full length *pkmps3* sequences.

Analysis on the diversity parameters and natural selection of members in Groups 1 and 2 was also carried out (Table 3). Haplotype diversity (Group 1: 0.993; Group 2: 0.995) and nucleotide diversity (Group 1: 0.02276; Group 2: 0.02418) of both groups were quite similar, as was the average number of nucleotide differences (Group 1: 24.31; Group 2: 25.97). The F_ST_ value between the groups was 0.402, indicating high genetic differentiation between these two groups. However, analysis of the phylogenetic tree did not indicate any temporal distribution between the two groups.

**Table 3:** Estimates of DNA diversity and selection for Group 1 and Group 2, which are the major clusters obtained in the phylogenetic analysis

*Pkmsp3*	H	Hd ± SD	π ± SD	K	Tajima’s D
Group 1	26	0.993 ± 0.011	0.02276 ± 0.00167	24.31	−0.81373
Group 2	19	0.995 ± 0.018	0.02418 ± 0.00226	25.37	−0.46858

*Abbreviations*: *H* number of haplotypes, *Hd* haplotype diversity, *K* average number of nucleotide differences, *π* nucleotide diversity, *SD* standard deviation

### Tests of selection for *pkmsp3*

Tests were carried out to determine if the diversity in *pkmsp3* was due to natural selection. The Tajima’s D, Fu & Li’s D* and F* tests showed no significant departure from neutrality in the full length *pkmsp3,* Domain A or Domain B (Table 1), thus suggesting neutral selection may be acting on these regions. Similarly, Tajima’s D test carried out on Group 1 and 2 showed no significant departure from neutrality (Table 3). This was reinforced by estimation of the d_N_/d_S_ ratio, where, the d_N_/d_S_ ratio for the full length sequence as well as Domain A were just slightly above 1, indicating neutral selection. However, the d_N_/d_S_ ratio for Domain B was 0.6, suggesting that this domain may be under purifying selection.

## Discussion

Vaccine development against malaria parasites is not a straightforward procedure. Multistage vaccines have recently been proposed because unique antigens are produced during the different stages of the parasite’s life-cycle. The merozoite has been identified as an important vaccine target due to its mobile and invasive nature, which exposes this stage to the host’s immune responses [42]. Many of the merozoite surface proteins contain polymorphic domains that signify diversifying selection, and conserved domains which indicate functional constraints of the protein. Furthermore, different strains within a *Plasmodium* species have been found to co-exist [43], thus vaccine candidates would need to be strain-transcending as one particular antibody generated against the protein from one strain may be ineffective against another. Antigenic diversity in vaccine candidates is one of the hurdles to design effective malaria vaccine. In vaccine development, it is prerequisite to survey genetic polymorphism of the candidate antigens, particularly the polymorphism from a wide range of field isolates. Furthermore, genetic polymorphism is also an important epidemiological tool. *Plasmodium knowlesi* has emerged in south-east Asia within the recent decade, and molecular epidemiological investigation may explain reasons of this recent emergence.

Although the biological functions of *P. vivax* and *P. knowlesi* MSP3 are not fully understood at this juncture, the alanine-rich central core in both proteins is predicted to form a coiled-coil tertiary structure [18]. Being located on the surface of the merozoites, the *P. vivax* MSP3 has been suggested to interact with other merozoite surface proteins, possibly mediated through protein-protein interactions involving the coiled-coil structure [18, 19] which is similar to what is observed in *P. falciparum* MSP3 [44]. In the present study, the coiled-coil region of *P. knowlesi* MSP3 was observed to be conserved. Therefore, similar to *P. falciparum* and *P. vivax* MSP3, the *P. knowlesi* MSP3 coiled-coil region may also utilise protein-protein interaction type bonds to interact with other merozoite surface proteins.

The nucleotide diversity (π: 0.046 ± 0.011) was found to be high when compared to other *P. knowlesi* functional genes [39–41], considering that most of the haplotypes discovered in this study were unique. A similar observation has also been reported for other merozoite surface antigens such as *eba175*, and this suggests that even where functional constraints exist, a range of haplotypes can still occur [45]. The low nucleotide diversity in Domain A as compared to that of the full length sequence, suggests limited polymorphism in the domain due to the presence of the coiled-coil region. Sliding window plot analysis (Fig. 2) showed high nucleotide diversity in the C-terminal, a finding also reported in *pvmsp3β* [20]. Temporal distribution of the haplotypes was not detected and this may be due to the fact that the *P. knowlesi* isolates were recent and collected within a 4-year period (2008–2012). The possibility of temporal distribution happening within such a short time is unlikely.

The *pkmsp3* gene shares significant homology with the *P. vivax pvmsp3* [46]. A study on *pvmsp3* of *P. vivax* isolates from Korea revealed nucleotide diversity of 0.0727 ± 0.002 and 0.0304 ± 0.001 at the N- and C-terminal domains respectively [47], which contrast the nucleotide diversity of *pkmsp3* domains (N-terminal π: 0.039 ± 0.002; C-terminal π: 0.067 ± 0.025). However, similar to *pkmsp3*, the C-terminal of *pvmsp3* had ratio of d_N_/d_S_ < 1, indicating purifying selection in that region. A study on *pvmsp3* of *P. vivax* isolates from Thailand found nucleotide diversity of 0.0877 ± 0.005 [48], which is comparatively higher than the nucleotide diversity of *pkmsp3* (π: 0.046 ± 0.011). Like *pkmsp3*, the C-terminal of *pvmsp3* of the Thailand isolates also showed purifying selection (d_N_/d_S_ < 1).

Phylogenetic and haplotype network analyses revealed that the *P. knowlesi* MSP3 haplotypes were clustered into two main groups. The Domain A in particular contributed to this clustering (Fig. 6). To gain a clearer picture of selection, the *Z*-test and Tajima’s D test for all three sets of sequences were analysed. In this instance, results for both the Z-test and Tajima’s D were not significant for the full length gene, Domain A or Domain B, indicating neutral selection. The d_N_/d_S_ ratio is widely used to evaluate the effect of natural selection on genes where a lack of d_N_ relative to d_S_ (d_N_/d_S_ < 1) suggests negative or purifying selection. Conversely, a higher value of d_N_ compared to d_S_ (d_N_/d_S_ > 1) is indicative of positive selection. The d_N_/d_S_ ratio for the full length gene as well as Domain A marginally exceeded 1, indicating neutral selection. Domain B, however, had a ratio of 0.6, indicating purifying selection on this part of the gene. Thus, it could be postulated that the *P. knowlesi* MSP3 has a functionally restricted Domain A which is protected from immune responses by an exposed and polymorphic Domain B.

In the present study, the phylogenetic tree showed separation of the *P. knowlesi* MSP3 haplotypes into two groups. Studies on *P. knowlesi* proteins such as the Duffy binding protein (PkDBPαII) [39], Pknbpxa [49] and PkAMA-1 domain I [50] have also reported bifurcation of haplotypes, indicating dimorphism of the genes. These findings provide support to the notion that two distinct *P. knowlesi* types or lineages exist in south-east Asia [51]. Microsatellite DNA analysis revealed two divergent *P. knowlesi* populations which have been associated with different macaque reservoir host species [52]. Recently, a whole-genome population study highlighted two major subgroups of *P. knowlesi* clinical isolates [53].

## Conclusions

To the best of our knowledge, the present study is the first to investigate genetic diversity of the *pkmsp3* gene as well as the natural selection acting on it. A moderate level of genetic diversity was observed in the *pkmsp3* and only the C-terminal region (Domain B) appeared to be under purifying selection. The separation of the *pkmsp3* into two groups of haplotypes provides further evidence of the existence of two distinct *P. knowlesi* types or lineages. Future studies should investigate the diversity of *pkmsp3* among *P. knowlesi* isolates in North Borneo, a region with reports of the highest number of human knowlesi malaria cases to date.

## Additional file

Additional file 1: Table S1. Multiple alignment of full amino acid sequences of *pkmsp3*. The yellow columns are the variable amino acid positions. The region highlighted in red at the top of the alignment indicates Domain A, and the region in green indicates Domain B. (XLS 389 kb)
